# Disruption of a Conservative Motif in the C-Terminal Loop of the KCNQ1 Channel Causes LQT Syndrome

**DOI:** 10.3390/ijms23147953

**Published:** 2022-07-19

**Authors:** Maria Karlova, Denis V. Abramochkin, Ksenia B. Pustovit, Tatiana Nesterova, Valery Novoseletsky, Gildas Loussouarn, Elena Zaklyazminskaya, Olga S. Sokolova

**Affiliations:** 1 Faculty of Biology, Lomonosov Moscow State University, 119234 Moscow, Russia; mkarlova@yandex.ru (M.K.); abram340@mail.ru (D.V.A.); k_pustovit@mail.ru (K.B.P.); valery.novoseletsky@yandex.ru (V.N.); 2 Institute of Immunology and Physiology, Ural Branch of Russian Academy of Sciences, 620049 Ekaterinburg, Russia; tatiannesterova@gmail.com; 3 Institute of Natural Sciences and Mathematics, Ural Federal University, 620075 Ekaterinburg, Russia; 4 Biology Department, Shenzhen MSU-BIT University, Shenzhen 517182, China; 5 Nantes Université, CNRS, INSERM, l’institut du Thorax, F-44000 Nantes, France; gildas.loussouarn@univ-nantes.fr; 6 Petrovsky National Research Center of Surgery, 119991 Moscow, Russia; zhelene@mail.ru

**Keywords:** KCNQ1, Kv7.1, I_Ks_, patch-clamp, inherited channelopathy, LQTS

## Abstract

We identified a single nucleotide variation (SNV) (c.1264A > G) in the KCNQ1 gene in a 5-year-old boy who presented with a prolonged QT interval. His elder brother and mother, but not sister and father, also had this mutation. This missense mutation leads to a p.Lys422Glu (K422E) substitution in the Kv7.1 protein that has never been mentioned before. We inserted this substitution in an expression plasmid containing Kv7.1 cDNA and studied the electrophysiological characteristics of the mutated channel expressed in CHO-K1, using the whole-cell configuration of the patch-clamp technique. Expression of the mutant Kv7.1 channel in both homo- and heterozygous conditions in the presence of auxiliary subunit KCNE1 results in a significant decrease in tail current densities compared to the expression of wild-type (WT) Kv7.1 and KCNE1. This study also indicates that K422E point mutation causes a dominant negative effect. The mutation was not associated with a trafficking defect; the mutant channel protein was confirmed to localize at the cell membrane. This mutation disrupts the poly-Lys strip in the proximal part of the highly conserved cytoplasmic A–B linker of Kv7.1 that was not shown before to be crucial for channel functioning.

## 1. Introduction

Potassium currents I_Ks_, I_Kr_, I_t0_, I_K1_, I_SS_, and I_K2P_ all contribute to repolarization in cardiomyocytes in normal and failing hearts [1]. Mutations in genes encoding the alpha and beta subunits of the potassium channels conducting these currents lead to several arrhythmic disorders, such as Long QT syndrome (LQTS), Short QT syndrome (SQTS), familial atrial fibrillation (FAF), Brugada syndrome (BrS), and early repolarization syndrome (ERS), and can also be found in cardiac sudden death (SCD) victims (OMIM). Loss-of-function mutations (LoF) usually lead to LQTS [2,3,4], whereas gain-of-function (GoF) mutations have a more variable appearance (SQTS, BrS, ERD, and FAF) [4,5]. Presumably, the clinical phenotype and the changes in ECG in patients with mutations in these genes correlate with ion permeability defects. On the other hand, there are many mixed and overlapping phenotypes, resulting from complex molecular pathways of channel dysfunction. Modern new-generation sequencing (NGS) technologies provide a unique opportunity to simultaneously test multiple genes in patients with suspected channelopathies and to identify the genetic cause of the disease [6]. However, the large volume of performed genetic testing reveals many rare/unique genetic variants of unknown clinical significance. The correct interpretation of these genetic findings is crucial for correct genetic counseling and clinical care, including the selection of the best choice of anti-arrhythmic treatment, SCD risk estimation, and decision-making about anti-arrhythmic device implantation. Hence, functional studies of newly discovered variants are critical to classify the variant as pathogenic or non-pathogenic.

KCNQ1 is a gene encoding the alpha subunit of the voltage-gated cardiac K^+^ channel complex, Kv7.1 (also named KCNQ1). This subunit assembles with the auxiliary beta subunit KCNE1 to form functional channel and generate the slowly activating delayed rectifier K^+^ current, I_Ks_. This current plays an important role in the repolarization of cardiac cells and the maintenance of the normal duration of cardiac action potentials. Mutations in the KCNQ1 gene are most common in patients with LQTS, the most common cardiac channelopathy [6]. Congenital LQTS is characterized by prolonged ventricular repolarization manifesting through QTc prolongation on surface ECG, and a high risk of polymorphic ventricular tachycardia, self-terminating or causing sudden cardiac death [7].

**Figure 1:**
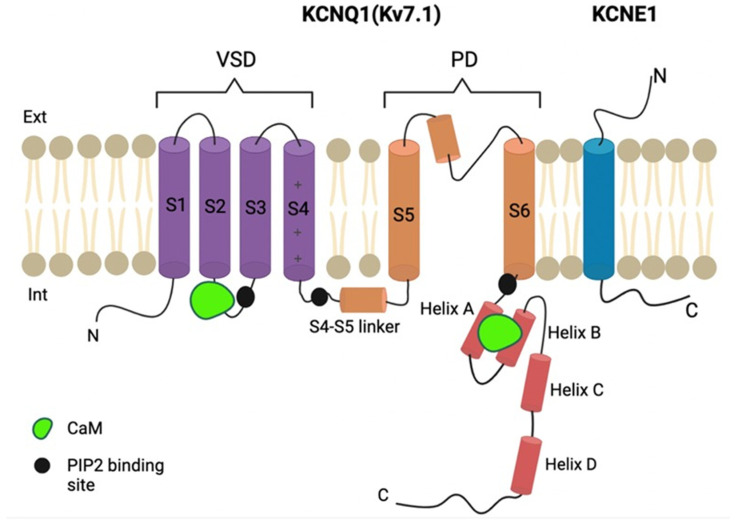
Schematic diagram of Kv7.1 ion channel organization. One alpha (KCNQ1) and one beta (KCNE1) subunit are shown. VSD—voltage-sensing domain; PD—pore domain; CaM—calmodulin.

The Kv7.1 ion channel family lacks the T1 tetramerization domain and does not associate with the cytoplasmic beta subunit, as in *Shaker* family channels [8,9]. Rather, it has a large intracellular C-terminal domain, ranging from 320 to 500 residues in size, bound constitutively to calmodulin (CaM) [10] (Figure 1). The C-terminal cytoplasmic domain is responsible for channel tetramerization, proper channel trafficking, and the maintenance of biophysical properties [11]. It comprises A, B, C, and D helices that are interconnected by A–B and B–C linkers [12]. A and B helices mediate binding to CaM [13]. The A–B linker is presumably unstructured, and this highly conserved loop has been predicted to be susceptible to proteolysis.

Previous electrophysiological experiments demonstrated that the A–B linker is not crucial for the correct functioning of Kv7 channels [14,15] and, thus, it was removed in various structural studies [13,16]. Recently, the structure of Kv7.1 with bound auxiliary subunit KCNE3, phospholipid phosphatidylinositol (4–5) bisphosphate (PIP_2_), and CaM was solved by cryo-EM [10], which revealed conformational changes in CaM and the cytoplasmic A subunit upon binding of PIP_2_.

Here, we identified a new single nucleotide variation (SNV) c.1264A > G (NM_000218.2) in a patient with LQTS. This variant is located in an A–B unstructured linker. The aim of this study was to perform a detailed biochemical and electrophysiological characterization of the Kv7.1-K422E mutant channel and to assess the possible role of this LoF mutation in QT prolongation.

## 2. Results

### 2.1. Clinical Case

**Figure 2:**
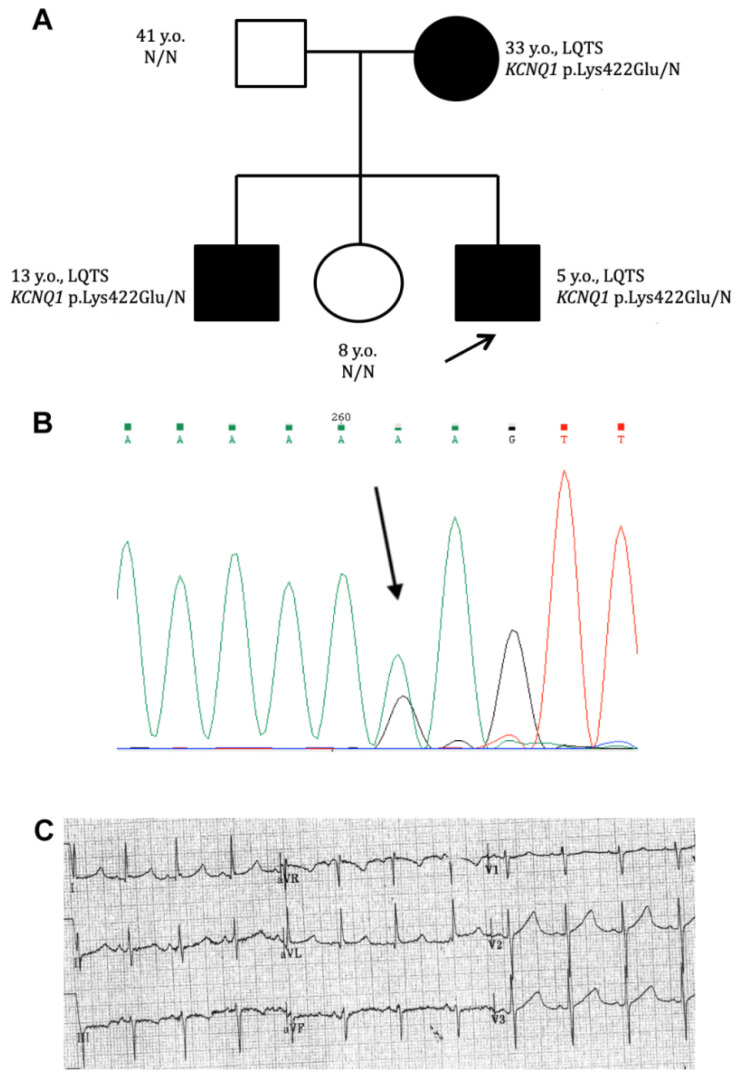
(**A**) Pedigree of family with genetic variant c.1264A > G (K422E) in the KCNQ1 gene. Family members with QTc prolongation—closed symbols; healthy individuals—open symbols. Arrow—proband. (**B**) Confirmation of the heterozygous c.1264A > G variant in the KCNQ1 gene by Sanger sequencing. Arrow—a.a. substitution. (**C**) Fragment of the proband’s ECG (5-year-old male, standing). Sinus rhythm, HR, 102 bpm, PQ, 142 ms, QTc, 570–587 ms.

Asymptomatic QTc prolongation (QTc 480 ms resting, and up to 587 ms in ortho probe; Bazett formula was employed) was registered in a 5-year-old male proband from a consanguineous family of Daghestani origin during a routine ECG screening (Figure 2A).

The familial history was unremarkable, and no SCD event or syncope in the family was disclosed by proband’s parents. A further clinical evaluation detected asymptomatic QTc prolongation in the proband’s mother (33 years old, QTc 489–495 ms) and his 13-year-old brother (QTc 478–485 ms). Proband’s sister (8 years old) and proband’s father (41 years old) had QTc within normal range (419 and 424 ms, respectively). Targeted gene panel testing revealed a rare non-characterized genetic variant c.1264A > G (p.Lys422Glu) in the KCNQ1 gene in the proband’s DNA (Figure 2B). The variant was assessed as a variant of unknown significance (VUS) according to ACMG2015 criteria. A familial genetic investigation detected two more carriers of this variant (mother and brother), both with QTc prolongation (Figure 2).

### 2.2. Expression and Subcellular Localization of Wild-Type and Mutant Kv7.1 Channels

**Figure 3:**
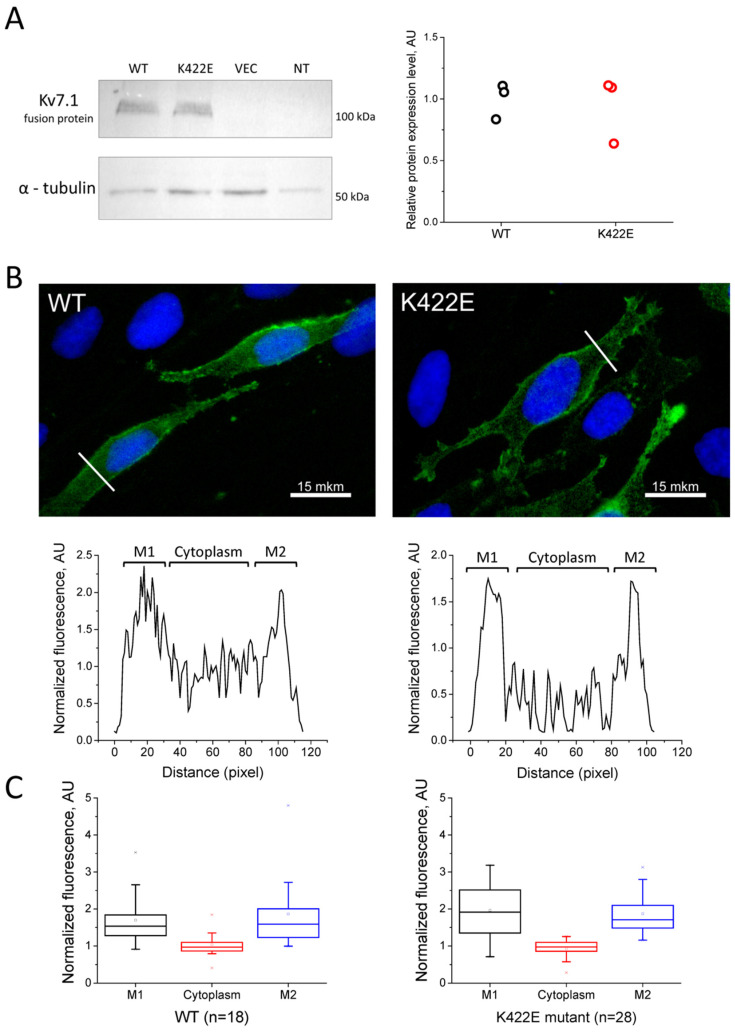
(**A**) Immunoblot analysis of Kv7.1 expression in whole-cell lysates of CHO-K1 cells. WT—cells transfected with control Kv7.1 protein; K422E—cells, transfected with Kv7.1 protein with K422E mutation; VEC—cells transfected with an empty vector; NT—non-transfected cells. Primary antibodies against V5-tag and alpha-tubulin. (*n* = 3 blots from three transfections). (**B**) Immunofluorescent staining of CHO-K1 cells expressing the recombinant Kv7.1 protein: wildtype (top left) and mutant (top right). Primary antibody against V5-tag, secondary antibody conjugated to alexa−488 dye (green channel). Nuclei are stained with DAPI (blue channel). Bottom—intensity of green signal across the cell (along the white lines in the images in the top row) with the two peaks corresponding to cell plasma membrane (M1 and M2). (**C**) Statistical analysis of the surface plots of Kv7.1 fluorescence intensity signal, mean ± SE fluorescence of Kv7.1 (WT, 18 cells, left; and K422E mutant, 28 cells, right) normalized to the average cell fluorescence intensity signal.

To check if the point mutation affects the Kv7.1 channel expression, the protein expression level was analyzed by immunoblot. The point mutation was introduced into the KCNQ1 cDNA, cloned into the appropriate vector, and expressed in CHO-K1 cells. An equal number of cells (50,000) were resuspended in PBS (10 μL) and loaded onto polyacrylamide gel. Alpha-tubulin was used as a loading control. Immunoblot analysis (Figure 3A) showed no differences in the Kv7.1 protein expression level in whole-cell lysates of CHO-K1 cells expressing wild-type (WT) or the K422E mutant.

To determine the effect of the mutation on the membrane expression of the Kv7.1 channel, fluorescent microscopy of CHO-K1 cells expressing Kv7.1-WT and Kv7.1-K422E subunits was performed. Analysis of the signal intensity across the cell (Figure 3B, below) confirmed membrane expression for both WT and mutant channels. As shown in plots on Figure 3C, both channel proteins showed substantial membrane localization. These data indicate that the K422E mutation does not disturb the expression and membrane trafficking of the mutant Kv7.1 protein.

### 2.3. Electrophysiological Properties of WT and K422E Channel Complexes

**Figure 4:**
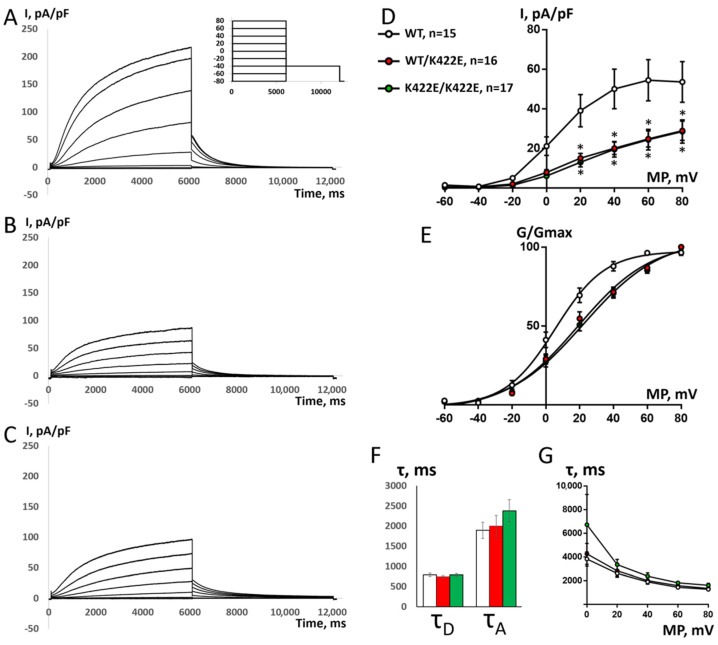
Electrophysiological properties of IKs current in transfected CHO-K1 cells. (**A**–**C**) Original recordings of the current from 3 different representative cells transfected with plasmid containing KCNE1 cDNA and either the WT Kv7.1 cDNA (**A**), or the same cDNA with introduced K422E mutation (**B**) or 1:1 combination of these two plasmids (**C**). The current was elicited by the 2-step square-pulse depolarization (shown in the inset), from the holding potential of −80 mV. The second step to −40 mV was used to analyze the outward tail current. (**D**–**G**) Comparison of the main parameters of IKs recorded from cells transfected with plasmid containing the WT Kv7.1 cDNA (*n* = 15), the same cDNA with introduced K422E mutation, homozygous K422E/K422E (*n* = 17), or 1:1 combination of these two plasmids (heterozygous WT/K422E, *n* = 16). The data is mean ± SEM. (**D**) I-V curves of peak tail IKs current in 3 groups of cells. * Significant difference from WT group, *p* < 0.05, two-way ANOVA with Tukey’s posthoc test. (**E**) Activation curves of IKs in 3 groups of cells. G/Gmax is the ratio between actual and maximal conductance. (**F**) Comparison of tail current deactivation kinetics (τD) after +40 mV depolarization in 3 groups of cells and IKs activation kinetics at depolarizing step to +40 mV (τA). (**G**) comparison of IKs activation kinetics in three groups of cells at potentials ranging from 0 to +80 mV.

A typical slow delayed rectifier K^+^ current (IKs) with slow activation and without any visible inactivation was elicited in transfected CHO-K1 cells by a 6 s depolarizing pulse from the holding potential of −80 mV to the potentials from −20 to +80 mV, with 20 mV steps (Figure 4A). The mutant current was significantly downregulated for both heterozygote WT/K422E (Figure 4B) and homozygote K422E/K422E groups (Figure 4C).

To determine the voltage dependence of activation, a series of depolarizing pulses were applied during 6 s, ranging from −60 to +80 mV, in 20 mV steps, for WT/WT, WT/K422E, and K422E/K422E groups (Figure 4A–C). Tail current densities measured at −40 mV were plotted as a function of the pre-pulse potential (Figure 4D). In the WT group, IKs’ current activation curve was almost saturated at +60 and +80 mV, while in both the K422E/K422E and WT/K422E groups, saturation was still not reached. Both WT/K422E and K422E/K422E showed a rightward shift of the activation curve (Figure 4E) indicated by a more positive V50 in comparison with the WT group (Supplementary Table S1). The slope factors were significantly larger in both WT/K422E and K422E/K422E groups in comparison with the WT group (Supplementary Table S1). However, no significant differences of V50 and slope factor were found between K422E/K422E and WT/K422E. The superposition of K422E/K422E and WT/K422E I/V curves and activation curves shows that K422E mutation exerts a dominant negative effect on WT subunit; otherwise, the WT/K422E activation curve would have been situated between the WT/WT and the K422E/K422E activation curve. At +60 mV, both the activation kinetics (τA) and tail current deactivation kinetics (τD) did not show any difference between WT, K422E/K422E, and WT/K422E groups (Figure 4F).

### 2.4. Modeling of the Influence of the K422E Mutation on Simulated Human Ventricular AP

Next, to evaluate the impact of Kv7.1 on the LoF, we performed mathematical simulations. To assess the effect of the K422E mutation on the AP waveform, we used the ionic model of human ventricular AP (epicardial version) TP2006 [17]. The model includes parameters of all major ionic currents of a human ventricular myocyte (see Section 4, Methods). Since the model was successfully used in numerous studies [18,19,20], it could be considered as one of the most reliable models for electrical activity of the non-ischemic human ventricular myocyte.

**Figure 5:**
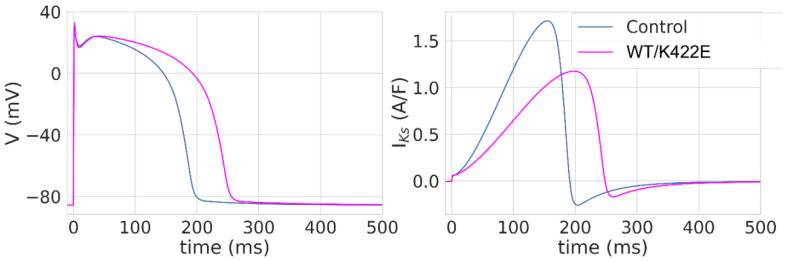
Simulated action potential (**left** panel) and IKs current (**right** panel) in control (WT) and heterozygote (WT/K422E) mutation models.

The comparison of simulated APs and related AP-induced IKs current traces is shown in Figure 5. The K422E mutation leads to marked AP prolongation at 50% and 90% repolarization levels without substantial changes in AP amplitude and resting membrane potential (Supplementary Table S2). The AP-induced IKs traces are characterized by slower activation and a lesser maximal value (Figure 5). Thereby, the results of our simulation study allow us to propose the slowing of repolarization induced by the K422E mutation in human ventricular cardiomyocytes, leading to the prolongation of the QT interval and the provocation of ventricular tachyarrhythmias.

## 3. Discussion

### 3.1. K422E Mutation Analysis

In this study, a toddler LQTS proband was found to have SNV c.1264A > G in the *KCNQ1* gene, resulting in p.Lys422Glu (K422E) amino acid substitution at the cytoplasmic C-terminus tail of the alpha-subunit of the IKs channel. This unique genetic variation has not been registered or characterized earlier. Two different allelic variants, p.Lys422Arg (K422R, single allele in gnomAD genomes, total MAF 0.00003191) and p.Lys422Thr (K422T, two alleles in gnomAD exomes, total MAF 0.000007917), were registered in gnomAD (https://gnomad.broadinstitute.org; accessed on 7 February 2022). The variant found within this study was classified as a variant of unknown significance (VUS, Class III) and, at the moment of detection, could not be used for diagnosis confirmation. The segregation analysis revealed this SNV in the two additional family members (older brother and mother) that also had asymptomatic QTc prolongation (Figure 2A). The sister and father had no QTc prolongation, nor did they carry this variant in their DNA.

To clarify the relationship between the K422E variant and the clinical phenotype, we inserted this substitution into the channel sequence and performed a detailed experimental investigation. The influence of this variant on the total expression level or on membrane distribution was ruled out by performing immunofluorescent analyses. Electrophysiological experiments showed that the K422E-Kv7.1 mutation results in the decrease of the K^+^ current and the alteration of channel activity (Figure 4).

We demonstrate that the exogenously expressed Kv7.1-K422E alpha subunit is sufficient for the disruption of Kv7.1 channel function (Figure 4), even when co-expressed with the WT form of the protein, suggesting that K422E is a dominant negative mutation. We obtained experimental evidence that this variant significantly alters channel permeability and can be characterized as a LoF mutation.

Numerous studies of C-terminal mutations in the KCNQ1 gene have previously shown that they often cause trafficking defects [21,22,23]. The mutation described here was not associated with a trafficking defect, since the mutant channel protein was detected at the cell membrane, similarly to the WT channel (Figure 3B,C).

According to prior studies [24], a majority of mutations (70%) that lead to LQTS are located in the pore region and in transmembrane domains S5 and S6 that are immediately adjacent to the pore, and the rest (30%) are in the N- and C-termini. The pore region mutations appeared to result in a more severe phenotype. On the contrary, patients with the K422E mutation located at the C-terminus showed a mildly affected phenotype.

Thus, we obtained experimental evidence that this variant significantly alters channel permeability and can be characterized as a LoF mutation. This is a classical mechanism of KCNQ1 mutations, leading to prolonged repolarization, which we clinically observe as a QTc prolongation on resting ECG. Thereby, functional data are in accordance with the clinical presentation (LQTS) in the family, and we can re-classify this variant as a “likely pathogenic” (Class IV) with PS3 (experimental in vitro evidence), PM2 (rarity in population), and PP3 (9 in silico predictors are in favor of pathogenicity) ACMG2015 criteria.

### 3.2. Proposed Mechanisms of Functional Loss

Here, we identified the single positive amino acid substitution to a negatively charged one, which led to a dramatic shift in I–V curves toward positive potentials (Figure 4). This shift suggests the presence of defective voltage sensing. A model that describes the voltage sensing of the Kv7.1 channel is a ligand/receptor model suggesting a loose coupling between the voltage sensors and the pore in Kv7.1 [25]: when the voltage sensors are resting, the S4–S5 linker binds to the S6 C-terminus as a ligand to its receptor, stabilizing the pore in a closed conformation; and during the activation, S4 drags the S4–S5 linker away from S6, releasing the pore. This model agrees with the experimental data not only for Kv7.1, but also cardiac Kv11.1 and neuronal Kv10.2 channels [26,27,28]. In these experiments, designed peptides that share a similar amino acid sequence with the S4–S5 linker (the ligand) of Kv7.1, Kv10.2, and Kv11.1 channels were co-expressed with the full-length channel, which resulted in a reduction of the voltage-dependent potassium current. One possibility would be that the unstructured A–B linker in the Kv7.1 channel, which comprises 120 residues, could possibly span the distance of up to 35 nm in an extended conformation that is sufficient to traverse the length between the voltage sensor and the cytoplasmic C-terminus of the channel.

**Figure 6:**
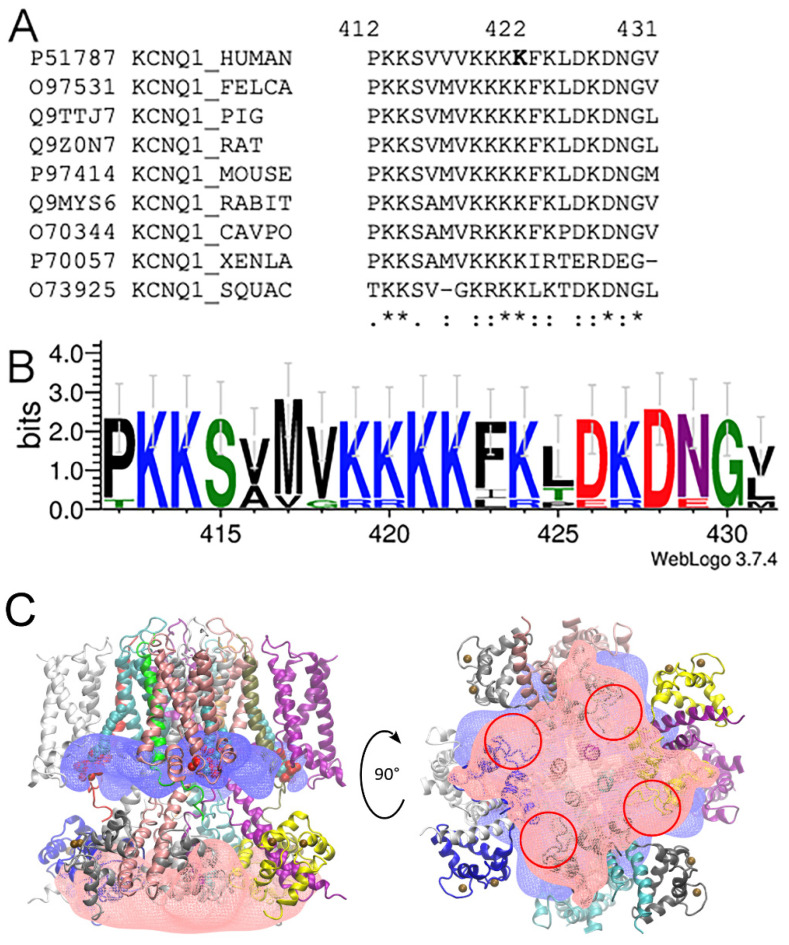
(**A**) Multiple sequence alignment for the 412–431 fragments of the human Kv7.1 and corresponding fragments of their relative sequences. Asterisks mark fully conserved positions. (**B**) Sequence logo for the fragment of an alignment of several Kv7.1 sequences. The height of each residue is proportional to its frequency, while the height of the overall stack of residues is inversely proportional to Shannon entropy. (**C**) Electrostatic potential maps showing positive (blue) and negative (red) potential near the Kv7.1-KCNE3-CaM-PIP_2_ complex. Blue and red electrostatic potential maps correspond to +400 kT/e and −400 kT/e, respectively. Protein chains are colored according to their IDs. Four PIP_2_ molecules are shown in space-filling representation. Eight Ca^2+^ ions are shown with brown spheres. Red circles correspond to the regions with the highest values of negative potential.

Another possibility is that introducing negatively charged Glu into position 422 apparently disrupts a conserved poly-Lys strip (Figure 6A,B) and may lead to the alteration of electrostatic interactions with other regions of the channel. This hypothesis agrees well with the fact that the substitution of Lys at the same position to the polar Thr residue did not affect the currents in the mutant channels, as compared to the WT [29].

In order to have more insights into the possible pathophysiological mechanism, we modeled the electrostatic potentials in the cytoplasmic area of the Kv7 channel (Figure 6C) by using the cryo-EM 3D reconstruction of a partially truncated (N- and C-termini were removed) Kv7.1 channel with bound CaM, auxiliary subunit KCNE3, and PIP_2_ lipids [10] (pdb id 6v01).

Post-translational modifications (PTM) of CaM were introduced according to Uniprot (https://www.uniprot.org/uniprot/P0DP23#ptm_processing; accessed on 30 January 2022), which lead to the net negative charges of −19 for every subunit of the cytoplasmic domain (+3 for fragment 355–390 and +10 for fragment 500–570 of Kv7.1, and −32 for CaM with PTM), which are partially neutralized by 8 Ca^2+^ ions (two of four possible for every CaM molecule). High values of the net charge generate substantial negative potential in the cytoplasmic domain, and we propose that this region (especially CaM molecules) could be a target for KKKKFK motif binding. The most negative regions of the potential are located in the vicinity of residues 100 and 135, which belong to Ca^2+^-binding motifs; however, in the current structure (pdb id 6v01), ions were absent in these sites. On the other hand, these regions are at the interface between CaM molecules (Figure 6C) formed with residues D79, D81, E83, E84, E85, and E88 of one molecule and residues D96; phosphorylated Y100; phosphorylated S102, D132, and D134; and phosphorylated Y139, E140, and E141 of the other (Supplementary Figure S1). We proposed that a positively charged KKKKFK motif could bind to this interface and stabilize it. Mutation K422E substantially decreases the charge of this motif and, hence, its ability to stabilize the CaM–CaM interface, which may lead to a repulsion of channel subunits in the cytoplasmic domain and to the disruption of channel function.

Moreover, this motif may also be needed for S-nitrosylation of Cys445 [30], as well as for binding antipsychotic drugs norfluoxetine and trifluoperazine [31,32]. Indeed, a study [31] of a patient with the K422T mutation who has elevated serum levels of norfluoxetine and was presented with Torsades de pointes (TdP) suggested the impaired channel functioning. More experiments, including possible pull-down assays, are needed to establish these interactions.

## 4. Materials and Methods

### 4.1. Clinical and Genetic Evaluation

A clinical and genetic evaluation was performed in accordance with the principles of the Declaration of Helsinki and with the written informed consent of adult family members. Instrumental examination included general examination, collection of personal and family history, general biochemical blood tests, resting and standing ECG 24 h Holter ECG monitoring, and echocardiography (EchoCG). A genetic study for proband was performed on DNA samples isolated from venous blood leukocytes, according to standard protocol. An NGS of the target panel of 11 genes (KCNQ1, KCNH2, KCNJ2, KCNE1, KCNE2, SCN5A, SCN1B-4B, and SNTA1) was performed based on the Ion Torrent PGM platform, using the Ion AmpliSeq™ Exome Kit (Thermo Fisher Scientific, Waltham, MA, USA). Primary data processing was performed by using Ion Proton Software (Thermo Fisher Scientific, Waltham, MA, USA); alignment with the hg19 version DNA reference sequence was performed by using the BWA 0.7.9 [33]. The pathogenicity of the identified variants was assessed by using the ACMG2015 pathogenicity criteria [31]. All rare (MAF < 0.01%) variants found by NGS were confirmed by capillary bidirectional Sanger re-sequencing on the ABI 3730XL DNA Analyzer (Thermo Fisher Scientific), according to manufacturer protocol. Cascade genetic testing for the family members was also performed by using the same instrument.

### 4.2. Plasmids

For Kv7.1 protein expression, two constructs were used. The first one—pcDNA6-V5-HisA/hKCNE1-hKCNQ1—contains the human KCNE1 auxiliary subunit fused to the human Kv7.1 alpha-subunit, inserted in frame with V5 and 6xHis tags in a pcDNA6-V5-HisA vector (Invitrogen Carlsbad, CA, USA). The second—plasmid pIRES2-EGFP/hKCNQ1-1D4—contains the human Kv7.1 alpha-subunit cDNA augmented with a 1D4-tag on the C-terminus of the polypeptide chain inserted in the pIRES2-EGFP vector (Clontech, Takara Bio, Mountain View, CA, USA). Plasmid construction was described earlier [34]. These plasmids were used as WT control and for mutagenesis. For electrophysiological experiments, the pIRES2-EGFP/hKCNQ1-1D4 (WT and K422E mutant) plasmids were used. For fluorescent microscopy, the pcDNA6-V5-HisA/hKCNE1-hKCNQ1 (WT and K422E mutant) plasmids were used, since the eGFP expression of the pIRES-EGFP vector interferes with the immunofluorescent staining. The construction of the pRC-KCNE1 plasmid encoding the KCNE1 auxiliary subunit was described elsewhere [35].

### 4.3. Introduction of a Point Mutation into a Channel Sequence

Mutations were introduced into plasmids pcDNA6-V5-HisA/hKCNE1-hKCNQ1 and pIRES2-EGFP/hKCNQ1-1D4, using site-directed mutagenesis. Single-point substitution was added to a forward primer. Non-overlapping reverse primers were used according to the polymerase manufacturer recommendations. Oligonucleotide AAAGAAAAAAGAGTTCAAGCTGGAC was used as a forward primer, and oligonucleotide ACCACCACAGACTTCTTG was used as a reverse primer. PCR was conducted with Q5 High-Fidelity polymerase in the presence of a GC-enhancer (Neb, Ipswich, MA, USA). Mutagenesis was verified by sequencing.

### 4.4. Cell Culture and Transfection

Cells were incubated in DMEM/F12 medium (Gibco, Thermo Fisher Scientific, USA) with 10% fetal bovine serum (HyClone, Marlborough, MA, USA), 2 mM glutamine, 100 U/mL penicillin, and 100 mg/mL streptomycin (Thermo Fisher Scientific, USA), in a CO2 incubator, at 37 °C.

A mixture of 0.5 µg pIRES2-EGFP/hKCNQ1-1D4 with cDNA encoding the Kv7.1 alpha-subunit, 0.5 µg pRC-KCNE1 with cDNA for the KCNE1 subunit, and 0.1 µg pMAX with cDNA for the green fluorescent protein (GFP) was co-transfected into CHO-K1 cells growing on round 35 mm Petri dishes, using Lipofectamine^®^ LTX with Plus™ Reagent (Thermo Fisher Scientific, USA). While the control group of cells (WT) was transfected with plasmid containing wild-type alpha-subunit cDNA, the “homozygous” experimental group (K422E/K422E) was transfected with plasmid containing mutant cDNA. The “heterozygous” experimental group (WT/K422E) was co-transfected with 0.25 µg wild-type and 0.25 µg mutant plasmid. All groups of cells were co-transfected with plasmid containing KCNE1 cDNA. The total amount of all DNA was the same in all experiments. Cells were incubated for 24 h and then seeded for electrophysiological recordings, which were performed 48–54 hafter transfection.

For fluorescent microscopy, cells were transiently transfected with plasmid pcDNA6-V5-HisA/hKCNE1-hKCNQ1, using Metafectene PRO (Biontex, Munich, Germany), according to manufacturer recommendations. Cells were seeded on glass coverslips in 35 mm plates 24 h before transfection. Plasmid DNA was mixed with Metafectene PRO (1:2), and transient transfection was performed when the cells were at 50% confluence. Cells were incubated in a complete medium without antibiotics for 48 h and then fixed for immunostaining.

### 4.5. Fluorescent Microscopy

Forty-eight hours after the transfection, the cells were fixed with 1.5% paraformaldehyde, permeabilized with 0.1% triton X-100 in PBS, blocked with 1% BSA in PBST, and stained with anti-V5-tag primary antibodies (Bio-Rad, Hercules, CA, USA). Second antibodies used were donkey anti-mouse Alexa 488 conjugated antibodies (Abcam, Waltham, MA, USA). Nuclei were stained with DAPI.

To quantify the ion channel expression in the membrane and cytoplasm, the intensity profile across the width of the cell was generated. This profile was segmented into 3 different sections: approximately 15% at each end of the cell profile, corresponding to the plasma membrane (M1 and M2, as shown in Figure 2B, lower graphs), and the remaining intermediate 70% of the surface plot, corresponding to the cytoplasmic compartments. For each of these areas in each cell, the Kv7.1 fluorescence intensity values were averaged and normalized to the average cell fluorescence intensity signal (*p* < 0.05 for membrane versus cytosol, paired Student’s *t*-test).

### 4.6. Immunoblotting

Mutant protein expression was analyzed by SDS–PAGE and immunoblotting with anti-1D4 and anti-V5 antibodies. The expression level was estimated by using ImageLab software (BioRad, Hercules, CA, USA). 

### 4.7. Electrophysiology

CHO-K1 cells on a small coverslip were placed in an experimental chamber with a constant flow of physiological solution of the following composition (mmol/L): 150 NaCl, 5.4 KCl, 1.8 CaCl_2_, 1.2 MgCl_2_, 10 glucose, and 10 HEPES; pH adjusted to 7.4 with NaOH; placed on the stage of an Eclipse Ti-S (Nikon, Tokyo, Japan) inverted fluorescence microscope. Currents were recorded at room temperature (24 ± 0.5 °C), using an Axopatch 200B amplifier (Molecular Devices, San Jose, CA, USA). Only cells emitting green fluorescent light when irradiated with 480 nm excitation light were selected for recording. Patch pipettes of 1.5–2.5 MOhm resistance were made of borosilicate glass (Sutter Instrument, Novato, CA, USA) and filled with the following solution (mmol/L): 140 KCl, 1 MgCl_2_, 5 EGTA, 4 MgATP, 0.3 Na_2_GTP, and 10 HEPES; pH was adjusted to 7.2 with KOH. Series resistance and pipette and cell capacitances were compensated before the start of each recording.

Current amplitudes were normalized to the capacitive cell size (pA/pF). The voltage dependence of activation (evaluated from normalized tail current amplitudes) was fitted with a Boltzmann equation, y = Imax/(1 + exp((V50 − V)/k)), to determine the membrane potential for half-maximal activation (V50) and the slope factor (k). Time courses of activation and deactivation were fitted with a single exponential function to obtain the time constants of activation (Τa) and deactivation (ΤD).

### 4.8. Computer Simulations of Human Ventricular Action Potential

The ionic model of human ventricular action potential (AP) (epicardial version) TP2006 [17] was used to assess the effect of the K422E mutation on the AP waveform. This model includes parameters of all major ionic currents of a human ventricular myocyte: fast Na^+^ current I_Na_, L-type Ca^2+^ current with fast and slow voltage inactivation, background inward rectifier K^+^ current I_K1_, transient outward current Ito, rapid delayed rectifier K^+^ current I_Kr_, slow delayed rectifier K^+^ current I_Ks_, Na^+^/Ca^2+^ exchanger current I_NCX_, currents of Ca^2+^ and Na^+^/K^+^ pumps, and background Na^+^ and Ca^2+^ currents. Intracellular calcium dynamics is represented by a single-compartment sarcoplasmic reticulum; Ca^2+^-induced Ca^2+^ release is described with a Markov-state model for the ryanodine receptor.

To simulate the experimental traces of IKs activation and tail currents, the following IKs parameters of the original model were updated: maximum conductivity (gKs), kinetic parameters V50, and slope. The fitting of these parameters was carried out by the least-squares method. In the original TP2006 model, gKs was 0.392 mS/μF, V50 was −5 mV, and slope was 14 mV [17]. Here, the values of gKs, V50, and slope in the control model (WT Kv7.1) were changed to fit our observed data.

The CVODE solver [36] and the Myokit software package [37] were used to solve the equations of the ionic model. The stimulation frequency was 1 Hz, the AP signals were recorded after reaching a steady state. To reach the steady state, 100 APs were calculated. The following characteristics of the simulated AP were measured: AP duration at 50% (APD50) or 90% (APD90) repolarization, AP amplitude (APA), and resting membrane potential (RMP).

### 4.9. Statistics

The results are represented as means ± SEM. Normality of distribution and equality of variances were checked, and necessary transformation of variables (see above) were made before statistical testing. The density of tail IKs in different groups of cells at different levels of membrane potential were compared by using two-way ANOVA with Tukey’s multiple comparisons test. The parameters of the steady-state activation curve, kinetics of current activation and tail current deactivation were compared by using the non-paired *t*-test. The *p*-values < 0.05 were considered statistically significant.

### 4.10. Electrostatics

The protein electrostatic potential was generated with the PME electrostatics plugin (PMEPot), using 144 × 144 × 128 grid position counts (~1 Å spacing) [38]. PQR files were prepared with the use of the PDB2PQR web service [39].

## Supplementary Materials

The following supporting information can be downloaded at https://www.mdpi.com/article/10.3390/ijms23147953/s1.
