# Supplementary material for: Disruption of a Conservative Motif in the C-Terminal Loop of the KCNQ1 Channel Causes LQT Syndrome
Source: Int J Mol Sci. 2022 Jul 19;23(14):7953. doi: 10.3390/ijms23147953 (PMC9316142; doi:10.3390/ijms23147953)
Supplement: Supplementary file 1 [file ijms-23-07953-s001.zip › ijms-1790046-supplementary.pdf]

Table S1. The parameters of IKs activation curve in CHO-K1 cells of WT, K422E/K422E and WT/K422Et groups. (* – significant difference from the control group, non-paired t-test, $p<0.05$).

Group	V_{50}		Slope	
	mean	SEM	mean	SEM
WT/WT, n=15	5.56	1.86	14.7	1.76
WT/K422E, n=16	19.1*	2.36	21.6*	2.53
K422E/K422E, n=17	22.4*	2.67	22.8*	2.83

Table S2. Characteristics of simulated action potential in control and heterozygote WT/K422E mutation models

	Control	WT/K422E
APD50 (ms)	171	226
APD90 (ms)	192	249
APA (mV)	118.6	118.3
RMP (mV)	-85.7	-85.5

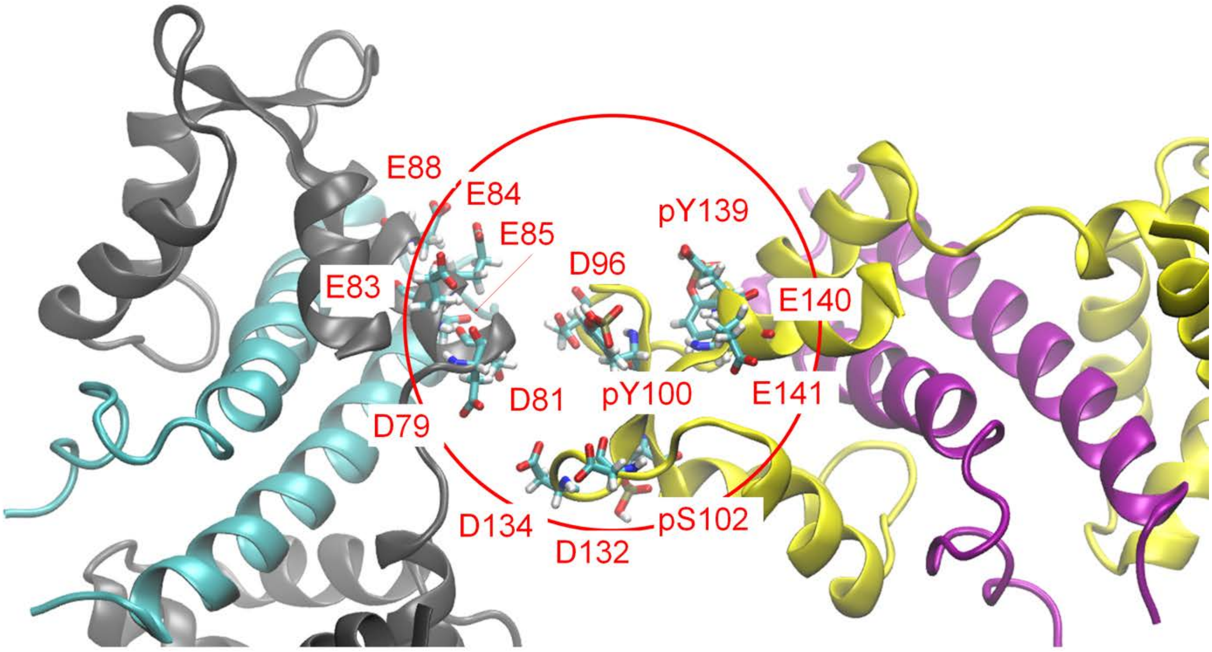

Figure S1. Fragment of KCNQ1-CaM complex. Protein chains are colored according to their IDs. CaM molecules shown in gray and yellow. Negatively charged residues at their interface shown in rod representation and subscribed. Red circle correspond to the region with the highest values of negative potential.
